# Could Malnutrition Be a Potential Parameter for Cardiac Risk Assessment in Older Adults?

**DOI:** 10.3390/jcm15062135

**Published:** 2026-03-11

**Authors:** Özge Özgün, Arzu Okyar Baş, Deniz Cengiz, Ceyda Kayabaşı, Aybüke Uyar, Okan Turhan, Arda Nacar, Cansu Çıkın, Murat Pehlivan, Cafer Balcı, Burcu Balam Doğu, Meltem Gülhan Halil, Mustafa Cankurtaran, Mert Eşme

**Affiliations:** 1 Division of Geriatric Medicine, Hacettepe University, Ankara 06230, Turkey; deniz.sahin232@gmail.com (D.C.); ceydakayaogullari@gmail.com (C.K.); aybuyar@outlook.com (A.U.); drokanturhan@gmail.com (O.T.); ardardanacar@gmail.com (A.N.); cansuckn@gmail.com (C.Ç.); pehlivanmmurat@gmail.com (M.P.); caferbalci@gmail.com (C.B.); bbdogu@gmail.com (B.B.D.); meltemhalil@gmail.com (M.G.H.); mustafacankurtaran@gmail.com (M.C.); mertesme87@hotmail.com (M.E.); 2 Ankara Education and Research Hospital, Ankara 06230, Turkey; arzu0506@hotmail.com

**Keywords:** malnutrition, cardiovascular risk assessment, older adults, major adverse cardiovascular events, mortality, mini nutritional assessment, frailty

## Abstract

**Background:** Malnutrition is highly prevalent in older adults and is associated with functional decline, systemic inflammation, and increased mortality. However, its prognostic role in relation to major adverse cardiovascular events (MACE), particularly when considered alongside established cardiovascular risk scores, remains insufficiently defined in geriatric populations. **Methods:** This retrospective cohort study included 291 adults aged ≥65 years who underwent a comprehensive geriatric assessment at a geriatric outpatient clinic. Nutritional status was evaluated using the Mini Nutritional Assessment—Short Form (MNA-sf). Cardiovascular risk was estimated using the Framingham Risk Score, SCORE2, SCORE2—Older Persons (SCORE2-OP), and LIFE-CVD (version 1). The primary outcome was the occurrence of MACE, and the secondary outcome was all-cause mortality. Multivariable logistic regression and Cox proportional hazards models were used to identify independent predictors of outcomes. **Results:** During a mean follow-up of 16.9 ± 4.7 months, 43 participants (14.8%) experienced MACE, and 11 (3.8%) died. Malnutrition or risk of malnutrition (MNA-sf < 12), present in 24.1% of participants, was significantly more frequent among those with MACE and those who died. In multivariable analyses, nutritional status remained a consistent independent predictor of both MACE and mortality, whereas commonly used cardiovascular risk scores showed limited or inconsistent associations with outcomes. **Conclusions:** In older adults, malnutrition assessed by the MNA-sf is a strong and independent predictor of both major adverse cardiovascular events and all-cause mortality, beyond traditional cardiovascular risk scores. These findings underscore the importance of incorporating nutritional status, together with frailty-related parameters, into cardiovascular risk assessment to improve risk stratification in geriatric care.

## 1. Introduction

Malnutrition, a global health problem resulting from insufficient, excessive, or imbalanced intake of energy or nutrients, is highly prevalent among older adults and is associated with physical and cognitive decline, reduced quality of life, and increased morbidity. In geriatric populations, malnutrition has been consistently linked to longer intensive care unit stays, higher readmission rates, increased infection risk, prolonged hospitalization, and elevated all-cause and cardiovascular mortality. Importantly, malnutrition represents both a cause and a consequence of disease in older inpatients, contributing to a vicious cycle of clinical deterioration [1,2,3,4].

The European Society for Parenteral and Enteral Nutrition (ESPEN) recommends the Mini Nutritional Assessment (MNA), in either its full or short form (MNA-sf), as the preferred screening tool for evaluating nutritional status in older adults. Previous studies have demonstrated that the MNA-sf is a reliable, age-specific instrument with good sensitivity and specificity for the identification of malnutrition and malnutrition risk in geriatric populations [5].

Beyond its impact on functional status, disease-related malnutrition is increasingly recognized as a contributor to systemic inflammation and cardiometabolic dysregulation. Inadequate nutritional intake may activate neuroendocrine and immune pathways, promote a pro-inflammatory milieu, impair antioxidant defences, and adversely affect vascular and myocardial function. Collectively, these mechanisms may contribute to increased cardiovascular vulnerability in older adults with poor nutritional status [5,6].

Large-scale cohort studies have led to the development of multivariable algorithms for estimating cardiovascular risk, which integrate multiple clinical parameters into a single score representing the probability of future cardiovascular events. These risk calculators are widely used in clinical practice to guide preventive strategies and therapeutic decision-making. However, despite the higher burden of cardiovascular disease in older populations, relatively few risk prediction models have been specifically developed or validated for geriatric patients, and their applicability in this age group remains limited [7,8,9].

In this context, the present study aimed to evaluate the association between nutritional status and all-cause mortality and major cardiovascular events in a geriatric population, while taking commonly used cardiovascular risk scores into account.

## 2. Materials and Methods

### 2.1. Study Design and Participants

This retrospective cohort study was conducted at the outpatient Geriatrics Clinic of Hacettepe University Faculty of Medicine, Ankara, Türkiye. The study included consecutive adults aged ≥65 years who attended the geriatric outpatient clinic between March 2021 and May 2024.

All patients underwent a comprehensive geriatric assessment (CGA) performed by a geriatrician during their baseline outpatient visit. Clinical, laboratory, and outcome data were subsequently retrieved retrospectively from the hospital information system and electronic health records.

Cardiovascular risk scores were calculated using baseline clinical and laboratory measurements obtained at the initial visit.

All CGA instruments—including the Mini Nutritional Assessment—Short Form (MNA-sf), Clinical Frailty Scale (CFS), Katz Activities of Daily Living (ADL), and Lawton–Brody Instrumental Activities of Daily Living (IADL)—were completed during the baseline outpatient visit. No repeated assessments of nutritional, functional, or frailty status were performed; therefore, all CGA data reflect baseline status at study entry [10,11,12,13,14,15,16].

Exclusion criteria were acute infection or inflammatory conditions within the preceding four weeks; acute cardiovascular events (acute coronary syndrome, stroke, or decompensated heart failure) within the previous three months; and missing data required for calculation of cardiovascular risk scores.

A consecutive sampling method was used, and all eligible patients during the predefined study period were included. Given the retrospective design, no formal a priori sample size calculation was performed; instead, the sample size was determined by the number of eligible patients during the predefined study period.

### 2.2. Baseline Assessments and Variables

Demographic characteristics (age and sex), medical history, and comorbidities (diabetes mellitus, hypertension, coronary artery disease, heart failure, atrial fibrillation, stroke, dementia, thyroid disorders, and chronic obstructive pulmonary disease), medication use, and lifestyle factors were recorded. Cancer history was documented as a binary variable irrespective of cancer type or disease activity.

Body mass index (BMI) was calculated as weight (kg) divided by height squared (m^2^). Laboratory parameters, including low-density lipoprotein cholesterol (LDL-C) and glycated hemoglobin (HbA1c), were obtained from baseline laboratory records.

Frailty was assessed using the 9-point Clinical Frailty Scale (CFS), with frailty defined as a CFS score ≥4. Functional status was evaluated using the Katz ADL and Lawton–Brody IADL scales, with higher scores indicating greater independence [10,11,12,13,14,15,16].

### 2.3. Nutritional Status

Nutritional status was assessed using the Mini Nutritional Assessment—Short Form (MNA-sf), a validated screening tool for older adults [16]. The MNA-sf score ranges from 0 to 14, with scores of 12–14 indicating normal nutritional status, 8–11 indicating risk of malnutrition, and ≤7 indicating malnutrition. For regression analyses, nutritional status was analyzed both as a continuous variable (MNA-sf score) and as a binary variable (malnutrition or risk of malnutrition defined as MNA-sf < 12).

### 2.4. Cardiovascular Risk Scores

The Framingham Risk Score (FRS) was used to estimate 10-year cardiovascular disease (CVD) risk based on established clinical variables from the Framingham Heart Study. Consistent with the traditional Framingham classification, participants were categorized as low (<10%), intermediate (10–19%), or high risk (≥20%) [17,18].

Systematic Coronary Risk Evaluation 2 (SCORE2) was calculated only in participants aged < 70 years, in accordance with guideline recommendations. Risk categories were defined as moderate (<5%), high (5% to <10%), and very high (≥10%). As all participants were of Turkish origin, the high-risk geographic region was applied [19].

For participants aged ≥70 years, cardiovascular risk was assessed using the SCORE2—Older Persons (SCORE2-OP) model. Risk categories were defined as low (<7.5%), medium (7.5–<15%), high (15–<30%), and very high (≥30%) [20].

Lifetime cardiovascular risk was assessed using the Life-Time Perspective Cardiovascular Disease (LIFE-CVD) prediction model (version 1). The LIFE-CVD model provides estimates of 10-year CVD risk (%) and cardiovascular disease-free life expectancy (years), derived within a competing-risk framework accounting for non-cardiovascular mortality. Both parameters were recorded as continuous variables. At the time of analysis, version 1 of the LIFE-CVD calculator was in use; the calculator was subsequently updated to version 2 [21].

### 2.5. Outcomes

The primary outcome was the occurrence of major adverse cardiovascular events (MACE), defined as non-fatal myocardial infarction, non-fatal ischemic stroke, coronary revascularization, hospitalization for acute heart failure, or new-onset atrial fibrillation. The secondary outcome was all-cause mortality.

### 2.6. Statistical Analysis

Categorical variables are presented as counts and percentages. Continuous variables are presented as mean ± standard deviation or median (interquartile range), as appropriate. Between-group comparisons were performed using the Student’s *t*-test or Mann–Whitney U test for continuous variables and the chi-square or Fisher’s exact test for categorical variables.

Multivariable logistic regression analyses were performed to identify independent predictors of MACE. To minimize collinearity with cardiovascular risk scores, age was dichotomised as ≥80 years. Separate models were constructed, including either a continuous MNA-sf score or malnutrition/risk of malnutrition (MNA-sf < 12), together with one cardiovascular risk score (FRS, SCORE2-OP, or LIFE-CVD) per model. Frailty and malnutrition are closely related and overlapping clinical syndromes in older adults; to avoid multicollinearity, CFS was excluded from primary multivariate models.

For mortality analyses, Kaplan–Meier survival curves were generated, and Cox proportional hazards regression models were used to estimate hazard ratios (HRs) and 95% confidence intervals (CIs). To evaluate the independent prognostic value of nutritional status beyond established cardiovascular risk scores, separate multivariable Cox models were constructed for each risk algorithm (Framingham Risk Score, SCORE2-OP, and LIFE-CVD). In each set of models, nutritional status was entered either as a continuous variable (MNA-SF score) or as a categorical variable (MNA-SF < 12), alongside the respective cardiovascular risk score. A two-sided *p* value < 0.05 was considered statistically significant. Statistical analyses were performed using IBM SPSS Statistics for Windows, Version 26.0 (IBM Corp., Armonk, NY, USA).

## 3. Results

The study included 291 older adults (37.8% men; mean age 73.7 ± 5.9 years). Frailty (CFS ≥ 4) was present in 147 participants (50.5%). The median MNA-sf score was 13 (IQR 12–14), and 70 participants (24.1%) were classified as malnourished or at risk of malnutrition (MNA-sf < 12) (Table 1).

### 3.1. Major Adverse Cardiovascular Events

During a mean follow-up of 16.9 ± 4.7 months, 43 participants (14.8%) experienced a major adverse cardiovascular event (MACE) (Table 2). MACE was more frequent in men and in participants with a history of coronary artery disease or stroke (all *p* = 0.001). Malnutrition or risk of malnutrition was more prevalent among participants with MACE (38.1% vs. 21.8%, *p* = 0.031), and LDL cholesterol levels were lower in this group (*p* = 0.043). Frailty tended to be more common among participants with MACE, although this did not reach statistical significance. Cardiovascular risk stratification using Framingham, SCORE2, SCORE2-OP, and LIFE-CVD (version 1) did not differ significantly between groups.

### 3.2. Mortality

During follow-up, 11 participants (3.8%) died (Table 3). Deceased participants were older, more frequently male, and had significantly poorer nutritional status, reflected by lower MNA-sf scores and a higher prevalence of malnutrition or risk of malnutrition (all *p* < 0.01). Low body weight was also associated with mortality (BMI < 20 kg/m^2^, *p* = 0.002). According to the Framingham classification, all deceased participants were categorized as high risk. In contrast, SCORE2-OP categories did not differ by mortality status. LIFE-CVD-derived 10-year cardiovascular risk estimates were lower among deceased participants (*p* = 0.031), while cardiovascular disease-free life expectancy did not differ significantly.

### 3.3. Survival Analysis

**Figure 1:**
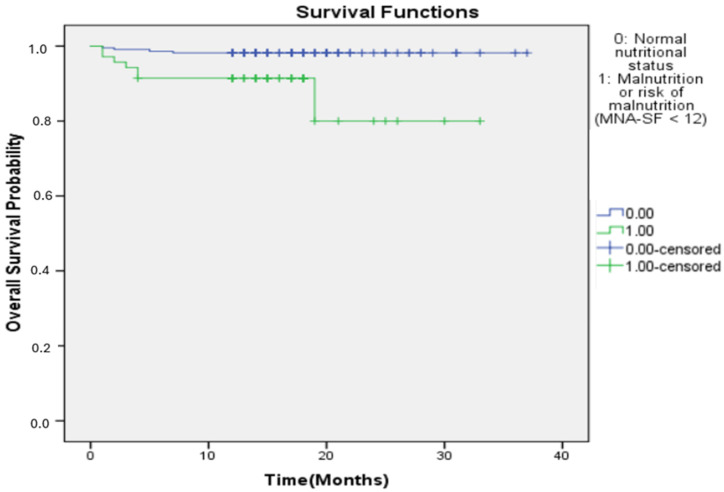
Kaplan–Meier survival curves for overall survival according to nutritional status assessed by the Mini Nutritional Assessment—Short Form (MNA-sf). Participants with malnutrition or risk of malnutrition (MNA-sf < 12) showed significantly lower overall survival compared with those with normal nutritional status (MNA-sf ≥ 12). Survival distributions differed significantly between groups (log-rank test, *p* = 0.001). Early separation of curves was also confirmed by the Breslow (generalized Wilcoxon) test, indicating increased short-term mortality among participants with impaired nutritional status.

Kaplan–Meier analysis demonstrated significantly lower survival among participants with malnutrition or risk of malnutrition compared with those with normal nutritional status. Mortality occurred in 10.0% of participants with malnutrition or malnutrition risk versus 1.8% of those with normal nutritional status. Median survival was not reached due to the low number of events (Figure 1).

### 3.4. Multivariable Analyses

Multivariable logistic regression analyses are shown in Table 4. Across all models, male sex and nutritional status assessed by MNA-sf were consistent independent predictors of MACE. In Framingham-adjusted models, each one-point increase in MNA-sf score was associated with reduced odds of MACE (OR 0.836, 95% CI 0.727–0.960; *p* = 0.011), while malnutrition or risk of malnutrition (MNA-sf < 12) was associated with an approximately 2.5-fold increased risk (OR 2.481, 95% CI 1.202–5.122; *p* = 0.014). Similar findings were observed in models adjusted for SCORE2-OP and LIFE-CVD-derived cardiovascular disease-free life expectancy.

Multivariable Cox regression models (Table 5) demonstrated that nutritional status, both as a continuous and categorical variable, was a predictor of mortality. Higher MNA-sf scores were consistently associated with lower risk across all models (Model 1A: HR 0.708; Model 2A: HR 0.732; Model 3A: HR 0.743; all *p* < 0.001)

Furthermore, participants categorized as having malnutrition or being at risk of malnutrition (MNA-sf < 12) exhibited a 4.2- to 5.2-fold increased risk compared with participants with normal nutritional status (*p* < 0.05). Notably, while the Framingham Risk Score remained significant in Model 1, other established risk calculators, such as SCORE2-OP and LIFE-CVD, did not maintain statistical significance when nutritional status was included in the models.

## 4. Discussion

In this study, the prognostic significance of nutritional status for cardiovascular outcomes and mortality in older adults was evaluated within the framework of a comprehensive geriatric assessment. Our findings demonstrate that individuals with malnutrition or at risk of malnutrition have a significantly higher risk of both major adverse cardiovascular events and all-cause mortality. In our cohort, malnutrition or risk of malnutrition was present in nearly one-quarter of participants and was consistently associated with adverse outcomes across univariable, multivariable, and survival analyses. These results highlight the importance of incorporating parameters that reflect biological reserve and frailty into geriatric risk assessment, beyond traditional cardiovascular risk factors.

Disease-related malnutrition is associated with multiple biochemical and physiological alterations, including reductions in serum albumin and prealbumin, total protein, transferrin, lymphocyte count, hemoglobin, muscle mass, and disturbances in lipid metabolism. These changes reflect depleted nutritional reserves, systemic inflammation, and catabolic stress, partly mediated by neuroendocrine and immune activation, resulting in increased pro-inflammatory cytokine release [19,22]. Inadequate intake of antioxidant micronutrients and an imbalance in dietary fatty acids may further promote oxidative stress and vascular inflammation, thereby impairing myocardial and vascular adaptability [5,6]. In line with these mechanisms, participants with MACE and those who died during follow-up in our study exhibited lower LDL cholesterol levels and poorer nutritional indicators, supporting the concept that malnutrition represents not merely a marker of poor general health but an active contributor to cardiovascular vulnerability and mortality in older adults.

In contrast to the strong and consistent associations observed for nutritional status, commonly used cardiovascular risk scores showed limited associations with outcomes in this geriatric cohort. Although all deceased participants were classified as high risk according to the Framingham Risk Score, SCORE2-OP categories were not significantly associated with either MACE or mortality. LIFE-CVD estimates demonstrated seemingly paradoxical patterns in both MACE and mortality analyses; however, this reflects the model’s age-dependent competing-risk structure. In older adults, higher non-cardiovascular mortality shortens the remaining time at risk for cardiovascular events, resulting in lower estimated lifetime cardiovascular risk despite substantial biological vulnerability. Therefore, lower LIFE-CVD estimates among participants with MACE or death are more likely to reflect limited remaining lifespan rather than reduced cardiovascular susceptibility, underscoring the need to interpret LIFE-CVD outputs within a geriatric context.

More broadly, the predictive performance of 10-year cardiovascular risk models declines with advancing age. Associations between traditional risk factors and cardiovascular disease weaken in older populations [21], and failure to adequately account for competing non-cardiovascular mortality may lead to overestimation of cardiovascular risk and treatment benefit. Moreover, cumulative atherosclerotic burden and prolonged exposure to risk factors may reduce the effectiveness of preventive interventions in late life [7,8,9,23,24,25]. Although SCORE2-OP was specifically developed for individuals aged ≥ 70 years, its lack of association with outcomes in our study may partly be explained by the limited sample size and number of events.

Frailty, which is not included in conventional cardiovascular risk models, represents a major limitation in geriatric risk assessment. Frailty reflects diminished physiological reserve and increased vulnerability to stressors and is strongly associated with mortality and disability [26]. In our cohort, more than half of the participants were living with frailty, and frailty was more prevalent among individuals with MACE and among those who died. Previous studies have shown that frailty is independently associated with increased cardiovascular risk, likely through shared inflammatory, hematological, and endocrine pathways [27]. Although current ESC guidelines emphasize the importance of frailty assessment in older adults, frailty remains absent from cardiovascular risk algorithms [28]. In the present study, frailty was not included in multivariable models because of its strong collinearity with nutritional status, highlighting the close interrelationship between these two geriatric syndromes and their overlapping contribution to biological vulnerability.

Key components of cardiovascular risk scores, including blood pressure and lipid parameters, may exhibit paradoxical patterns in older adults. Frail individuals often present with lower measured blood pressure despite a high prevalence of hypertension, reflecting impaired autonomic regulation, comorbidity burden, malnutrition, and treatment-related effects [29,30,31]. Likewise, age-related hormonal and inflammatory alterations, together with widespread statin use, may result in lower lipid levels independent of intrinsic cardiovascular risk. Consequently, low LDL cholesterol levels—traditionally considered cardioprotective—have been associated with increased mortality in frail and malnourished older adults, a phenomenon referred to as the lipid paradox [25,31,32]. Consistent with this concept, LDL cholesterol levels in our study were lower among participants who experienced MACE and among those who died, without evidence of a protective association, underscoring the limited interpretability of isolated lipid values in geriatric populations. These findings also have implications for lipid-lowering treatment strategies in older adults. The use of statins in older adults is an important clinical consideration in cardiovascular risk management. Current guidelines do not define an upper age limit for statin therapy; instead, treatment decisions should be individualized based on life expectancy, comorbidity burden, frailty, and potential drug interactions. Evidence suggests that statins can be beneficial in selected older patients, particularly in secondary prevention. However, in frail individuals with multimorbidity or limited life expectancy, the balance between potential benefit and adverse effects should be carefully evaluated [33,34,35].

Body weight represents another factor subject to reverse epidemiology in older adults. While low body weight is consistently associated with increased mortality, overweight and mild obesity may confer a survival advantage in geriatric populations [36,37,38]. In our cohort, low body weight and BMI < 20 kg/m^2^ were significantly more frequent among deceased participants, supporting the presence of an obesity paradox and further emphasizing the role of nutritional reserve in late-life outcomes.

Similarly, diabetes and glycemic control were not associated with MACE or mortality in our cohort. Although nearly half of the participants had diabetes mellitus, cardiovascular risk models performed poorly in this subgroup, which is consistent with previous reports highlighting the limitations of general population risk scores in individuals with diabetes. HbA1c demonstrates a U-shaped relationship with mortality, whereby both low and high values are associated with increased risk. Low HbA1c may reflect malnutrition and inadequate carbohydrate intake, whereas malnutrition-related insulin resistance may contribute to elevated HbA1c levels [39,40,41,42,43,44,45,46,47].

It should also be acknowledged that malnutrition in older adults may, at least in part, represent a consequence of underlying chronic disease burden, functional decline, or systemic inflammation rather than a purely causal factor. However, even when malnutrition reflects disease severity, its strong and independent association with adverse outcomes underscores its value as an integrative marker of biological vulnerability and reduced physiological reserve. From a clinical perspective, this dual role highlights the importance of nutritional assessment not only as a prognostic indicator but also as a potential target for intervention in geriatric care. In line with this necessity, nutritional interventions should be holistic, as outlined by the ESPEN (European Society for Clinical Nutrition and Metabolism) guidelines, aiming for an energy intake of 27–30 kcal/kg/day and a protein intake of at least 1.0–1.2 g/kg/day. These targets should be individualized based on the patient’s specific clinical context, such as renal function or acute illness. In addition to macronutrients, maintaining adequate hydration is crucial to mitigate additional cardiovascular and renal risks. Poor dental status and gastrointestinal symptoms, which are often overlooked, are fundamental determinants of successful nutritional management. Collaboration between geriatricians, dietitians, and caregivers is essential for achieving optimal nutritional outcomes. Caregivers play a vital role in preparing meals and ensuring adherence to nutritional plans and daily fluid intake [48].

Several limitations should be acknowledged. The retrospective, single-centre design limits causal inference and generalisability. Secondly, the small number of deaths reduced statistical power and increased the risk of model instability. A longer follow-up is needed to obtain more robust estimates. Thirdly, cancer history was recorded as a binary variable without stratification by disease activity, which may have resulted in residual confounding. Fourthly, malnutrition and risk of malnutrition were analyzed together using an MNA-sf cut-off of ≤12, which may have limited the ability to distinguish prognostic differences between these two categories. Fifthly, most participants were receiving medical therapy, which may have influenced both cardiovascular risk score estimates and clinical outcomes. Tools such as the Framingham Risk Score and SCORE2/SCORE2-OP are designed to estimate long-term (10-year) cardiovascular risk. However, the follow-up period in our study was shorter than this horizon, and therefore, longer-term follow-up studies are needed to better evaluate their predictive performance. Additionally, nutritional status was assessed only at baseline and may change over time. Subclinical or newly developed diseases during follow-up could have influenced both nutritional status and subsequent adverse outcomes. Changes in nutritional status were not recorded during the follow-up period. Finally, depression, socioeconomic status, and detailed medication use were not systematically assessed, which may have introduced additional residual confounding.

Despite these limitations, the present findings highlight the importance of incorporating nutritional assessment into cardiovascular risk evaluation in older adults. Malnutrition emerged as a strong and independent predictor of both MACE and mortality, whereas traditional cardiovascular risk scores showed limited discrimination in this population. These results support a comprehensive, multidimensional approach to cardiovascular risk assessment in geriatric patients, integrating nutritional status and frailty alongside conventional risk factors.

## 5. Conclusions

In older adults, malnutrition assessed by the Mini Nutritional Assessment—Short Form is a strong and independent predictor of both major adverse cardiovascular events and all-cause mortality. The prognostic value of nutritional status persists beyond commonly used cardiovascular risk scores, which showed limited discrimination in this geriatric cohort. Given their close interrelationship, malnutrition and frailty should be considered complementary components of cardiovascular risk assessment rather than isolated geriatric syndromes. Integrating nutritional status and frailty into routine cardiovascular risk evaluation may enable more accurate identification of vulnerable older adults and support a more comprehensive, person-centred approach to risk stratification in geriatric care.

## Figures and Tables

**Table 1:** The demographic properties and general characteristics.

Male, n(%)			110 (37.8%)
Female n (%)			181 (62.2%)
Age, mean ± SD			73.7 ± 5.9
CFS, median (IQR)			4 (3–4)
Living with frailty (CFS ≥ 4), n(%)			147 (50.5%)
CAD, n (%)			80 (27.5%)
Diabetes mellitus, n(%)			138 (45.5%)
Hypertension, n (%)			224 (73.9%)
Congestive heart failure, n (%)			19 (6.6%)
Stroke, n (%)			19 (6.6%)
Dementia, n(%)			18 (6.2%)
Cancer, n (%)			46 (15.9%)
MNA-sf score, Median (IQR)			13 (12–14)
Malnutrition and risk of malnutrition (MNA-sf < 12), n (%)			70 (24.1%)
Katz, median (IQR)			6 (5–6)
Lawton, median (IQR)			8 (6–8)
BMI ± SD			28.6 ± 5.9
Obesity (BMI > 30), n (%)			96 (38.9%)
BMI < 20, n (%)			9 (3.6%)
BMI < 22, n (%)			16 (6.5%)
LDL (mg/dL) ± SD			122.7 ± 35.8
HbA1C, median (IQR)			6.2 (5.8–7.0)
Framingham, score			17.0 (2–30)
Framingham Risk Score (10-year CVD risk)	Low risk, n (%)	FRS < 10%	25 (8.8%)
	Intermediate risk, n (%)	(FRS 10–19%)	79 (27.9%)
	High risk, n (%)	(FRS ≥ 20%)	179 (63.3%)
SCORE2	Moderate risk, n (%)	(<5%)	1 (1.2%)
	High risk, n (%)	(5% to <10%)	37(44.6%)
	Very high risk, n (%)	(≥10%)	45 (54.2%)
SCORE2-OP	Low risk, n (%)	(<7.5%)	41 (20.5%)
	Medium risk, n (%)	(7.5–<15%)	47 (23.5%)
	High risk, n (%)	(15–<30%)	75 (37.5%)
	Very high risk, n (%)	(≥30%)	37 (18.5%)
LIFE-CVD (version 1), CVD-free life expectancy (years)			24.8 ± 12.3
LIFE-CVD (version 1), 10-year CVD risk (%)			10.1 ± 5.9

CFS: Clinical Frailty Scale. CAD: Coronary artery disease. MNA-sf: Mini Nutritional Assessment. CVD: Cardiovascular disease. BMI: Body mass index. LDL: Low-density lipoprotein HbA1c: Glycated hemoglobin. SCORE2 was calculated only in participants aged < 70 years, in accordance with guideline recommendations (n = 83). LIFE-CVD estimates were interpreted within a competing-risk framework, accounting for non-cardiovascular mortality.

**Table 2:** Demographic characteristics of the participants according to the presence of major cardiovascular adverse events.

		Without MACE n = 248 (85.2%)	With MACE n = 43 (14.8%)	*p* Value
Men, n (%)		86 (34.7%)	24 (54.8%)	**0.001**
Age, mean ± SD		73.5 ± 5.9	75 ± 5.4	0.086
CFS, median (IQR)		4 (3.0–4.0)	4 (3.0–4.0)	**0.038**
Living with frailty (CFS ≥ 4), n (%)		120 (50.2%)	27 (65.9%)	0.081
CAD, n (%)		58 (23.4%)	22 (51.2%)	**0.001**
Diabetes mellitus, n (%)		118 (47.6%)	20 (46.5%)	0.897
Hypertension, n (%)		189 (76.2%)	35 (81.4%)	0.558
Congestive heart failure, n (%)		14 (5.7%)	5 (11.9%)	0.169
Stroke, n (%)		10 (4.0%)	9 (21.4%)	**0.001**
Cancer, n (%)		39 (15.8%)	7 (16.7%)	0.823
MNA-sf score, Median (IQR)		13 (12.0–14.0)	13 (10.0–14.0)	0.095
Malnutrition and risk of malnutrition (MNA-sf < 12), n (%)		54 (21.8%)	16 (38.1%)	**0.031**
Katz, median (IQR)		6 (5–6)	6 (5–6)	0.724
Lawton, median (IQR)		8 (6–8)	8 (6–8)	0.235
BMI ± SD		28.9 ± 5.4	28.4 ± 6.49	0.917
Obesity (BMI > 30), n (%)		84 (39.6%)	12 (34.3%)	0.581
LDL (mg/dL)		124.4 ± 34.9	113.7 ± 39.0	**0.043**
HbA1C, median (IQR)		6.20 (5.8–7.0)	6.1 (5.7–7.0)	0.965
Dementia, n (%)		16 (6.5%)	2 (4.7%)	1.000
Framingham score		17 (2–26)	18 (10–30)	0.294
Framingham Risk Score	Low risk, n (%) FRS < 10%	21 (8.7%)	4 (9.5%)	0.181
	Intermediate risk, n (%) (FRS 10–19%)	72 (29.9%)	7 (16.7%)	
	High risk, n (%) (FRS ≥ 20%)	148 (61.4%)	31 (73.8%)	
SCORE2	Moderate risk, n (%) (<5%)	1 (1.3%)	0 (0%)	0.606
	High risk, n (%) (5% to <10%)	34 (45.3%)	3 (37.5%)	
	Very high risk, n (%) (≥10%)	40 (53.3%)	5 (62.5%)	
SCORE2-OP	Low risk, n (%), (<7.5%)	33 (19.9%)	8 (23.5%)	0.541
	Medium risk, n (%) (7.5–<15%)	44 (26.5%)	3 (8.8%)	
	High risk, n (%) (15–<30%)	58 (34.9%)	17 (50%)	
	Very high risk, n (%) (≥30%)	31 (18.7%)	6 (17.6%)	
LIFE-CVD (version 1), CVD-free life expectancy (years)		25.2 ± 12.4	22.4 ± 11.3	0.192
LIFE-CVD (version 1), 10-year CVD risk (%)		10 ± 5.7	11 ± 7	0.670

MACE: Major adverse cardiovascular events. CFS: Clinical Frailty Scale. CAD: Coronary artery disease. MNA-sf: Mini Nutritional Assessment. CVD: Cardiovascular disease. BMI: Body mass index. LDL: Low-density lipoprotein. HbA1c: Glycated hemoglobin. SCORE2 was calculated only in participants aged <70 years, in accordance with guideline recommendations (n = 83). LIFE-CVD estimates were interpreted within a competing-risk framework, accounting for non-cardiovascular mortality. Bold values indicate statistically significant results (*p* < 0.05).

**Table 3:** Demographic characteristics of the participants according to mortality.

		Alive n = 280 (96.2%)	Dead n = 11 (3.8%)	*p* Value
Men, n (%)		102 (36.4%)	8 (70.0%)	**0.015**
Age, mean ± SD		73.5 ± 5.8	79.0 ± 5.7	**0.005**
CAD		75 (26.8%)	5 (45.5%)	0.181
Diabetes mellitus		134 (47.9%)	4 (36.4%)	0.454
Hypertension		215 (76.8%)	9 (81.8%)	1.000
Congestive heart failure		17 (6.1%)	2 (18.2%)	0.158
Stroke		17 (6.1%)	2 (18.2%)	0.158
Cancer, n (%)		43 (15.5%)	3 (27.3%)	0.294
MNA-sf score, median (IQR)		13 (12–14)	9 (8–12)	**0.004**
Malnutrition and risk of malnutrition (MNA-sf < 12)		63 (22.6%)	7 (63.6%)	**0.005**
Katz, median (IQR)		6 (5–6)	6 (4–6)	0.051
Lawton, median (IQR)		8 (6–8)	7 (2–8)	**0.038**
BMI ± SD		28.8 ± 5.7	24.8 ± 9.9	0.069
Obesity (BMI > 30), n(%)		93 (39.2)	3 (30.0)	0.745
CFS, median (IQR)		4 (3.0–4.0)	5.0 (4.0–7.0)	**0.001**
Living with frailty (CFS ≥ 4)		138 (49.3%)	9.0 (81.8%)	**0.034**
BMI < 20, n (%)		6 (2.8%)	3 (42.9%)	**0.002**
LDL (mg/dL)		123.5 ± 35.7	104.4 ± 33.6	0.064
HbA1c		6.1 (5.8–7.0)	6.40 (5.5–7.1)	0.908
Dementia		18 (6.5%)	0 (0%)	1.000
Framingham score		17 (2–26)	18 (15–30)	0.127
Framingham Risk Score	Low risk, n (%) FRS < 10%	25 (9.2%)	0 (0%)	**0.012**
	Intermediate risk, n(%) (FRS 10–19%)	79 (29.0%)	0 (0%)	
	High risk, n (%) (FRS ≥ 20%)	168 (61.8%)	11 (100%)	
SCORE2	Moderate risk, n (%) (<5%)	1 (1.2%)	0 (0%)	NA *
	High risk, n (%) (5% to <10%)	37 (44.6%)	0 (0%)	
	Very high risk, n (%) (≥10%)	45 (54.2%)	0 (0%)	
SCORE2-OP	Low risk, n (%),(<7.5%)	39 (20.6%)	2 (18.2%)	0.958
	Medium risk, n (%)(7.5–<15%)	46 (24.3%)	1 (9.1%)	
	High risk, n (%)(15–<30%)	67 (35.4%)	8 (72.7%)	
	Very high risk, n (%) (≥30%)	37 (19.6%)	0 (0%)	
LIFE-CVD (version 1), CVD-free life expectancy (years)		25.1 ± 12,3	14.9 ± 10.3	**0.031**
LIFE-CVD (version 1), 10-year CVD risk (%)		10 ± 5.79	11 ± 8.1	0.828

CFS: Clinical Frailty Scale. CAD: Coronary artery disease. MNA-sf: Mini Nutritional Assessment. CVD: Cardiovascular disease. BMI: Body mass index. LDL: Low-density lipoprotein. HbA1c: Glycated hemoglobin. SCORE2 was calculated only in participants aged < 70 years, in accordance with guideline recommendations (n = 83). LIFE-CVD estimates were interpreted within a competing-risk framework, accounting for non-cardiovascular mortality. Bold values indicate statistically significant results (*p* < 0.05). * *p* value could not be calculated because there were no events in the mortality group.

**Table 4:** Logistic regression analyses of the independent factors associated with the presence of the major cardiovascular adverse events.

		OR (95% CI)	*p*-Value
Model 1A	Age (≥80)	0.742 (0.283–1.942)	0.543
	Men, sex	2.209 (1.075–4.541)	**0.031**
	Cancer	0.949 (0.377–2.388)	0.911
	MNA-sf	0.834 (0.727–0.957)	**0.010**
	Framingham Risk Score	1.031 (0.937–1.135)	0.530
Model 1B	Age (≥80)	0.734 (0.280–1.923)	0.529
	Men, sex	2.238 (1.086–4.613)	**0.029**
	Cancer	1.007 (0.406–2.497)	0.988
	Malnutrition and risk of malnutrition (MNA-sf < 12)	2.515 (0.928–1.126)	**0.012**
	Framingham Risk Score	1.022 (0.919–1.118)	0.658
Model 2A	Age (≥80)	0.531 (0.175–1.610)	0.264
	Men, sex	2.171 (0.991–4.753)	**0.053**
	Cancer	0.945 (0.337–2.647)	0.914
	MNA-sf	0.834 (0.717–0.972)	**0.020**
	SCORE2-OP	1.014 (0.977–1.052)	0.460
Model 2B	Age (≥80)	0.541 (0.179–1.635)	0.276
	Men, sex	2.145 (0.983–4.479)	0.055
	Cancer	1.018 (0.372–2.790)	0.972
	Malnutrition and risk of malnutrition (MNA-sf < 12)	2.375 (1.050–5.371)	**0.038**
	SCORE2-OP	1.024 (0.974–1.047)	0.595
Model 3A	Age (≥80)	0.718 (0.254–2.031)	0.533
	Men, sex	1.928 (0.934–3.981)	0.076
	Cancer	1.027 (0.406–2.600)	0.955
	MNA-sf	0.863 (0.749–0.996)	**0.043**
	LİFE-CVD (CVD-free life expectancy (years))	1.020 (0.955–1.089)	0.556
Model 3B	Age (≥80)	0.706 (0.249–2.001)	0.513
	Men, sex	1.942 (0.940–4.012)	0.073
	Cancer	1.065 (0.425–2.667)	0.894
	Malnutrition and risk of malnutrition (MNA-sf < 12)	2.126 (0.976–4.630)	0.058
	LİFE-CVD (CVD-free life expectancy (years))	1.019 (0.953–1.089)	0.586

MNA-sf: Mini Nutritional Assessment—Short Form. CVD: Cardiovascular. SCORE2 was calculated only in participants aged < 70 years, in accordance with guideline recommendations (n = 83). LIFE-CVD estimates were interpreted within a competing-risk framework, accounting for non-cardiovascular mortality. Model 1A: Age (≥80), sex, cancer, MNA-sf, and Framingham Risk Score. Model 1B: Age (≥80), sex, cancer, malnutrition and risk of malnutrition (MNA-sf < 12), and Framingham Risk Score. Model 2A: Age (≥80), sex, cancer, and MNAS-f, SCORE2-OP. Model 2B: Age (≥80), sex, cancer, malnutrition and risk of malnutrition (MNA-sf < 12), and SCORE2-OP. Model 3A: Age (≥80), sex, cancer, MNA-sf, and LİFE-CVD CVD-free life expectancy (years). Model 3B: Age (≥80), sex, cancer, malnutrition and risk of malnutrition (MNA-sf < 12), and LİFE-CVD CVD-free life expectancy (years). Bold values indicate statistically significant results (*p* < 0.05).

**Table 5:** Multivariable Cox regression models evaluating the independent association between nutritional status and major adverse cardiovascular events (MACE).

		HR (95% CI)	*p* Value
Model 1A	MNA-sf	0.708 (0.583–0.859)	**0.001**
	Framingham Risk Score	1.158 (1.014–1.323)	**0.030**
Model 1B	Malnutrition and risk of malnutrition (MNA-sf < 12)	5.252 (1.528–18.05)	**0.008**
	Framingham Risk Score	1.153 (1.002–1.328)	**0.048**
Model 2A	MNA-sf	0.732 (0.602–0.890)	**0.002**
	SCORE2-OP	1.009 (0.961–1.059)	0.730
Model 2B	Malnutrition and risk of malnutrition (MNA-sf < 12)	5.218 (1.524–17.86)	**0.009**
	SCORE2-OP	0.996 (0.949–1.046)	0.872
Model 3A	MNA-sf	0.743 (0.611–0.903)	**0.003**
	LİFE-CVD (CVD-free life expectancy (years))	0.951 (0.887–1.020)	0.159
Model 3B	Malnutrition and risk of malnutrition (MNA-sf < 12)	4.201 (1.170–15.09)	**0.028**
	LİFE-CVD (CVD-free life expectancy (years))	0.942 (0.878–1.011)	0.095

MNA-sf: Mini Nutritional Assessment—Short Form CVD: Cardiovascular disease. SCORE2 was calculated in participants aged <70 years (n = 83), while SCORE2-OP was used for those aged ≥ 70 years (n = 108), in accordance with current guideline recommendations. LIFE-CVD estimates were interpreted within a competing-risk framework, accounting for non-cardiovascular mortality. Model 1A: MNA-sf, Framingham Risk Score. Model 1B: Malnutrition and risk of malnutrition (MNA-sf < 12) and Framingham Risk Score. Model 2A: MNA-sf and SCORE2-OP. Model 2B: Malnutrition and risk of malnutrition (MNA-sf < 12) and SCORE2-OP. Model 3A: MNA-sf and LİFE-CVD (CVD-free life expectancy (years)). Model 3B: Malnutrition and risk of malnutrition (MNA-sf < 12) and LİFE-CVD (CVD-free life expectancy (years)). Bold values indicate statistically significant results (*p* < 0.05).

## Data Availability

The data presented in this study are not publicly available due to ethical and privacy restrictions, as they contain sensitive clinical information. Data are available from the corresponding author upon reasonable request and with permission of the local Ethics Committee.
